# Impact of plasmapheresis on severe COVID-19

**DOI:** 10.1038/s41598-022-25930-8

**Published:** 2023-01-04

**Authors:** G. Fonseca-González, M. Alamilla-Sánchez, V. García-Macas, J. Herrera-Acevedo, M. Villalobos-Brito, E. Tapia-Rangel, D. Maldonado-Tapia, M. López-Mendoza, J. H. Cano-Cervantes, J. Orozco-Vázquez, D. Timarán-Montenegro, S. Cortés-Martínez, M. Escarela-Serrano, S. Muñoz-López, L. Montiel-López, P. Mondragón-Terán, J. A. Suárez-Cuenca

**Affiliations:** 1 Department of Nephrology, Transplantation and Extracorporeal Therapies, Centro Médico Nacional “20 de Noviembre” ISSSTE, Mexico City, Mexico; 2 Department of Internal Medicine, Centro Médico Nacional “20 de Noviembre” ISSSTE, Mexico City, Mexico; 3 Department of Therapeutic and Diagnostic Radiology, Centro Médico Nacional “20 de Noviembre” ISSSTE, Mexico City, Mexico; 4 Hormone Laboratory, Centro Médico Nacional “20 de Noviembre” ISSSTE, Mexico City, Mexico; 5 Clinical Research Department, Centro Médico Nacional “20 de Noviembre” ISSSTE, Mexico City, Mexico

**Keywords:** Physiology, Medical research, Nephrology

## Abstract

The clinical course of COVID-19 may show severe presentation, potentially involving dynamic cytokine storms and T cell lymphopenia, which are leading causes of death in patients with SARS-CoV-2 infection. Plasma exchange therapy (PLEX) effectively removes pro-inflammatory factors, modulating and restoring innate and adaptive immune responses. This clinical trial aimed to evaluate the impact of PLEX on the survival of patients with severe SARS-CoV-2 and the effect on the cytokine release syndrome. Hospitalized patients diagnosed with SARS-CoV-2 infection and cytokine storm syndrome were selected to receive 2 sessions of PLEX or standard therapy. Primary outcome was all-cause 60-days mortality; secondary outcome was requirement of mechanical ventilation, SOFA, NEWs-2 scores modification, reduction of pro-inflammatory biomarkers and hospitalization time. Twenty patients received PLEX were compared against 40 patients receiving standard therapy. PLEX reduced 60-days mortality (50% vs 20%; OR 0.25, 95%CI 0.071–0.880; p = 0.029), and this effect was independent from demographic variables and drug therapies used. PLEX significantly decreased SOFA, NEWs-2, pro-inflammatory mediators and increased lymphocyte count, accompanied with a trend to reduce affected lung volume, without effect on SatO_2_/FiO_2_ indicator or mechanical ventilation requirement. PLEX therapy provided significant benefits of pro-inflammatory clearance and reduction of 60-days mortality in selected patients with COVID-19, without significant adverse events.

## Introduction

Since December 2019, health workers and governments around the world have been fighting a virus that changed our lives. COVID-19 is an infection caused by the severe acute respiratory syndrome coronavirus type 2 (SARS-CoV-2)^1^. Until November 2021, around five million people had died globally as a result of COVID-19. The data recorded in America already exceeded 2.3 million deaths^2^.

Coronavirus disease is characterized by a wide spectrum of manifestations, ranging from asymptomatic to acute respiratory failure, multi-organ failure, and death, the result of macrophage activation and cytokine storm^3^. According to a report with more than 44,500 confirmed cases, up to 81% were mild disease (mild or nonexistent pneumonia); 14% were moderate diseases (eg dyspnea, hypoxia, or pulmonary involvement > 50% on tomography in the first 24–48 h); and cases of severe disease (respiratory failure, shock or multi-organ dysfunction) were reported in 5%^4^.

The severity of COVID-19 is associated with host response and increased release of inflammatory mediators including cytokines and chemokines such as interleukin (IL) -2, IL-6, IL-7, IL-10, tumor necrosis factor (TNF), C-reactive protein (CRP), ferritin, and D-dimer in blood after SARS-CoV-2 replication^5^, these mediators promote alveolar endothelial inflammation and acute respiratory distress syndrome (ARDS)^5–8^. Interleukin-6 level highly correlates with COVID-19 mortality, when COVID-19 survivors and non-survivors are compared^9^, suggesting that lethal COVID-19 is characterized by excessive cytokine release that can lead to a cytokine storm syndrome^10^.

Effective antiviral treatment and measures to modulate the innate immune response and restore the adaptive immune response are imperative to break the cycle and enhance the effect of treatment. Some drug therapies would take weeks or months to remove these pro-inflammatory factors, while plasma exchange therapy (PLEX) is able to effectively remove these large molecules^11^. This technique includes the removal of large plasma volumes, which must be replaced with replacement fluids (e.g., albumin, fresh frozen plasma)^12^. This also makes it possible to selectively eliminate substances of high molecular weight from the intravascular space or to replace a deficient circulating factor^13,14^.

The effectiveness of PLEX depends on the plasma volume (PV) removed from the patient, on the distribution of the pathogen to be removed. One exchange (1:1) is equivalent to 65% of the initial component removed from the intravascular space. The first session of PLEX, which is prescribed with 1.5 plasma volume, removes approximately 75% of target molecules from the intravascular compartment, while the second session achieves a removal of 85% of target molecules^15^.

This trial aimed to evaluate the impact of plasmapheresis therapy on the survival of patients with severe SARS-CoV-2 and cytokine release syndrome who received at least two sessions during their hospitalization compared against standard therapy.

## Methods

Study design. Our trial was designed to evaluate the impact of plasmapheresis therapy in the outcome of severe COVID-19 and cytokine release syndrome at one medical center in Mexico City. The study was designed and performed according to ethical guidelines of the 1975 Declaration of Helsinki, and approved by the Local Committees of Research, Ethics in Research and Biosafety of the Centro Médico Nacional ‘20 de Noviembre’ ISSSTE, Mexico City (Protocol ID No. 09-136.2021). All participants provided written informed consent.

Study population. Hospitalized patients between April-August 2020. Patients were aged 16 to 65 years old, diagnosed with SARS-CoV-2 infection, as confirmed by typical tomographic findings according to Radiological Society of North America and/or qRT-PCR confirmation. Clinical presentation of Acute Respiratory Distress Syndrome and increased levels of interleukine-6 > 40 pg/mL, ferritin > 500 ng/mL, C reactive protein (CRP) > 60 mg/L, erythrocyte sedimentation rate > 40 m/s; and/or lymphopenia < 1.0 × 10/L. Patients were excluded if they had SOFA score > 11 points, active bleeding, platelet count < 50,000 cels and/or hypofibrinogenemia < 80 mg/dL. Written informed consent was obtained from all the patients or from a legal representative if they were unable to provide consent.

Standard therapy. It was based on the use of chloroquine, azithromycin, dexamethasone, supplementary oxygen; as well as tocilizumab and intravenous immunoglobulin. This therapy was common for the control and PLEX group, while PLEX group received tocilizumab and immunoglobulin before PLEX therapy.

Plasmapheresis. A double lumen central venous catheter was placed, either at jugular or femoral vein approaches. The plasmapheresis therapy was performed with a membrane-based system, using PrismaFlex CRRT and a TPE 1000–2000 filter according to body surface. Exchange plasma volumes of 1.5 times the estimated circulating plasma volume, according to Kapplan’s formula (Plasma volume (lts) = 0.065 × Weight (kg)  ×  (1   Hematocrit [%])). The blood flow rate was set at range 75–150 ml/min. Replacement solution consisted of 3% albumin, at a flow rate started at 100 mL/h, and increased up to maximum of 1500 mL/h; while 2 fresh frozen plasma were transfused at the end of each session. Anticoagulation was performed at doses of 30–40 IU/Kg/h of unfractionated heparin. A second plasmapheresis session was systematically performed 48 h after the first session. Blood samples were obtained from catheter blood before and after every session, for cytokine determination.

Cytokine determination. Blood samples were centrifuged and 500 mcL of serum are used for cytokine determination. Briefly, serum was combined with surface-bound capture anti-IL6 polyclonal antibody and alkaline phosphatase system developer as tracer (IL6 Bead Pack, Diagnostic Products Corporation, LA Cal. USA), in a immulite 2000 automatized immunoassay (Siemens), working within a range of 3–870 pg/mL. This system works under certification ISO 13485:2003.

Lung damage determination. One day before starting treatment and ten days after the last PLEX, the volume of lung involvement was calculated through tomographic volumetric assessment. Non-contrast enhanced chest CT imaging was performed using 2 CT scanners (Siemens SOMATOM drive and Siemens SOMATOM emotion scanners, Siemens Healthineers, Germany). Imaging reconstructions were performed with a 1-mm thickness slices without interstice gap. Lung segmentation was performed using the Alma Medical workstation version 5.0. For determination of non-affected lung parenchyma volume (NLV), automated segmentation tool was selected, and a reference attenuation range between − 1000 and − 600 HU was designated. Vascular structures, airways, and pathologic opacities were excluded. For determination of Lung Opacities Segmentation-Lung Opacities Volume (LOV): Initial automated segmentation was attempted using thresholding-based methods. A reference attenuation range between − 500 HU and 20 HU was selected. Then, the semi-automated option was selected to perform a region-based segmentation to adjust lesion boundaries. Volumes from each side were added to calculate total NLV and LOV. Total lung volume was calculated adding NLV + LOV.

Outcomes. The primary outcome was all-cause mortality within 60 days after inclusion. Secondary outcomes were the free mechanical-ventilation days, changes in SOFA score, decrease of pro-inflammatory markers at day 7, hospital length-of-stay, and decrease of lung’s volume involvement.

Statistical analysis. Data distribution was assessed by Kolmogorov–Smirnoff test. Then, qualitative and quantitative data were resumed as n(%) and mean±SD, respectively. Inferential analyses were performed by either chi square, one-way independent T-test, or U-Mann–Whitney. Kaplan–Meyer curves were constructed. Risk estimation was evaluated through OR and CI 95%. Statistical significance was considered at p < 0.05.

### Ethics approval and consent to participate

The study was designed and performed according to ethical guidelines of the 1975 Declaration of Helsinki, and approved by the Local Committees of Research, Ethics in Research and Biosafety of the Centro Médico Nacional ‘20 de Noviembre’ ISSSTE, Mexico City (Protocol ID No. 09-136.2021). All participants provided written informed consent.

## Results

Twenty patients who were eligible to receive plasmapheresis (PLEX) constituted the study population, and were compared against 40 patients who only received standard therapy.

Patients in the PLEX group were mean aged 47 years old, 85% males, with obesity (80%) as most prevalent comorbidity; and significantly higher ferritin, IL-6 and lower platelet count, as compared to control group. There were no differences regarding baseline SOFA and NEWs-2 scores, or other pro-inflammatory, pro-coagulant proteins and/or acid/base equilibrium between PLEX and control group, as shown in Table 1.

**Table 1:** Baseline characteristics of the study population.

	Control (n = 40)	PLEX (n = 20)	P value
Male, n (%)	29 (72.5)	17 (85.0)	0.23
Age (years ± SD)	48.4 ± 11.2	46.7 ± 13.1	0.30
Comorbidities, n (%)			
Type 2 diabetes	12 (30.0)	4 (20.0)	
Systemic arterial hypertension	9 (22.5)	3 (15.0)	
Overweight/obesity [BMI > 24.9 kg/m^2^]	19 (47.5)	16 (80.0)	
Preexistent pneumopathy	9 (22.5)	6 (30.0)	0.40
Immunosuppression therapy, n (%)	2 (5)	4 (20.0)	0.08
Other therapy for SARS-CoV-2, n (%)			
Tocilizumab	12 (30.0)	9 (45.0)	0.19
Immunoglobulin IV	22 (55.0)	12 (60.0)	0.46
SOFA (points)	3.32 ± 2.72	4.65 ± 3.01	0.06
NEWs-2 (points)	7.82 ± 2.73	7.00 ± 2.90	0.28
SaO2/FiO2 (ratio)	258.00 ± 90.80	276.00 ± 46.60	0.20
Ferritin (ng/mL)	981.5 ± 119.6	2096.0 ± 422.4	< 0.01
Interleukine-6 (pg/mL)	40.50 (17.15–81.70)	129.50 (46.30–665.50)	< 0.01
ESR (m/s)	35.24 ± 2.3	40.1 ± 17.4	0.12
CRP (mg/L)	146.3 ± 94.4	176.3 ± 110.8	0.29
Plasma creatinine (mg/dL)	0.97 ± 0.59	1.67 ± 2.62	0.11
Lymphocytes (cel/mm), mean ± SD	1.20 ± 0.97	0.96 ± 0.53	0.12
Hemoglobin (g/dL), mean ± SD	14.81 ± 1.93	14.68 ± 2.47	0.93
Platelets (cells), mean ± SD	230 ± 84	208 ± 95	< 0.01
pH, mean ± SD	7.41 ± 0.07	7.42 ± 0.05	0.77
HCO_3_, mean ± SD	21.41 ± 2.80	22.03 ± 2.89	0.16
pCO2, mean ± SD	33.95 ± 9.45	34.28 ± 6.37	0.48
D dimer (µg/L), median (IQR)	1.10 (0.60–2.12)	0.95 (0.44–1.87)	0.40
Fibrinogen (mg/dL), mean ± SD	540.20 ± 173.70	623 ± 264.30	0.35

Qualitative data were expressed as n(%) and quantive data were expressed as mean  ±  standard deviation. Abbreviatures: PLEX, plasma exchange therapy; BMI, body mass index; SOFA, Sequential Organ Failure Assessment Score; CRP, C-reactive protein; ESR, erythrosedimentation rate.

Regarding time-to-PLEX, mean time to receive first exchange was 4.9 ± 3.1 days, and mean time from the first symptom to PLEX was 12.2 ± 5.2 days. During PLEX therapy, the mean volume exchange was 4.46 ± 0.37 l.

**Figure 1:**
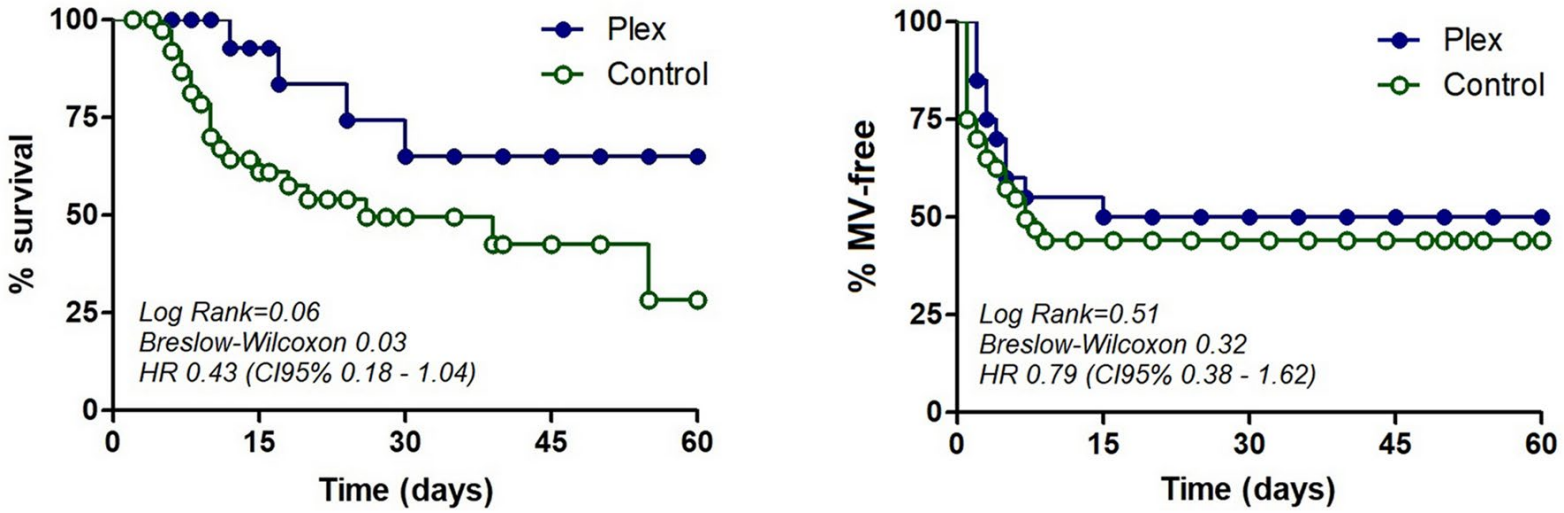
Effect of PLEX on mortality and mechanical ventilation—free time. Kaplan–Meier curves comparing % of survival (left) and % of patients without mechanical ventilation requirement.

**Figure 2:**
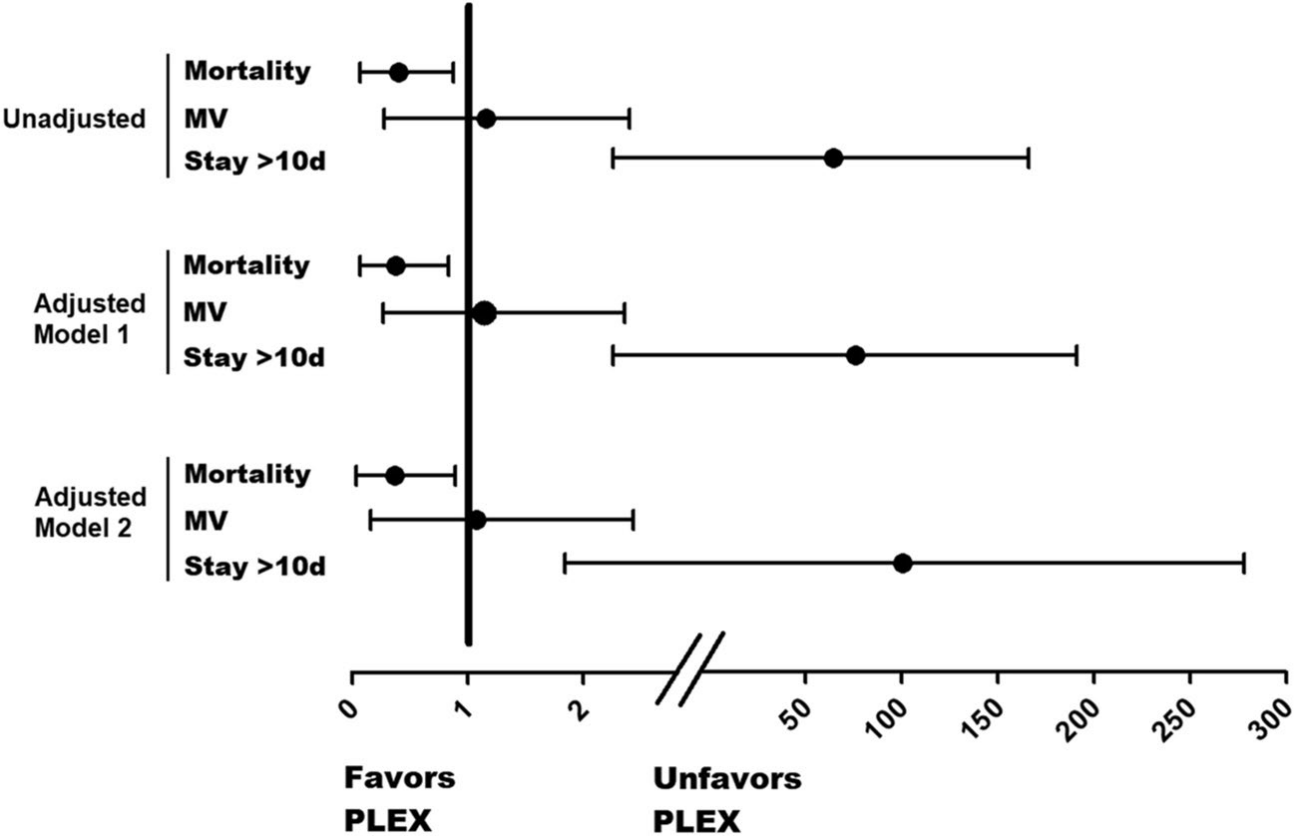
PLEX and risks of dead, mechanical ventilation and longer hospital stay. Forrest plot showing ORs corresponding to the effect of PLEX over dead, mechanical ventilation and longer hospital stay. Three models are shown, unadjusted (upper), adjusted model 1 (middle, adjusted by age and gender) and adjusted model 2 (lower, adjusted by age, gender and drug therapy including tocilizumab and immunoglobulin).

Regarding the primary outcome, PLEX group reduced 60-days mortality (PLEX 20% vs 50%; OR 0.25 95%CI 0.071–0.880; p = 0.029), and survival curves are shown in Fig. 1. Moreover, effect on mortality was independent from demographic variables and drug therapies used (Fig. 2). However, PLEX did not affect the risk of mechanical ventilation and it was associated with longer hospital stay. Regarding severity scores, SOFA and NEWs2 scores tended to decrease in PLEX group, with an opposite effect in the control group (p = 0.02, Table 2).

**Table 2:** Clinical, biochemical and tomographic characteristics before and after receiving PLEX.

	Before treatment	After treatment^a^	P value
**Clinical scores**			
SOFA score, median (IQR)			
Plasma exchange	4.65 ± 3.74	3.65 ± 0.01	0.18
Control	3.32 ± 2.72	5.17 ± 4.25	0.02
NEWs-2 score, median (IQR)			
Plasma exchange	7.00 ± 2.90	5.50 ± 2.42	0.06
Control	7.82 ± 2.73	6.38 ± 4.02	0.02
SaO_2_/FiO_2_, mean ± SD			
Plasma exchange	276.0 ± 46.60	271.20 ± 101.10	0.38
Control	258.0 ± 90.80	234.50 ± 120.10	0.07
**Biochemicals results**			
Ferritin (ng/mL), mean ± SD			
Plasma exchange	2096.0 ± 422.40	1019.0 ± 539.50	< 0.01
Control	981.50 ± 119.60	1531.0 ± 3110.0	0.37
Interleukine-6 (pg/mL), median (IQR)			
Plasma exchange	129.50 (46.30–665.50)	14.60 (9.41–50.20)	< 0.01
Control	40.50 (17.15–81.70)	12.00 (4.00–54.60)	0.02
Erythrocyte sedimentation rate (µm/s), mean ± SD			
Plasma exchange	40.10 ± 17.4	31.20 ± 16.3	0.05
Control	35.20 ± 12.20	40.50 ± 12.1	0.07
C-reactive protein (mg/L), mean ± SD			
Plasma exchange	176.0 ± 111	57.0 ± 71.40	< 0.01
Control	144.0 ± 98.20	122.0 ± 96.80	0.26
Creatinine (mg/dL), mean ± SD			
Plasma exchange	1.67 ± 2.61	0.85 ± 0.89	< 0.01
Control	0.97 ± 0.59	1.32 ± 1.50	0.22
Lymphocytes (cel/mm), mean ± SD			
Plasma exchange	0.96 ± 0.53	1.37 ± 1.03	0.05
Control	1.20 ± 0.97	1.91 ± 2.39	0.18
Hemoglobin (g/dL), mean ± SD			
Plasma exchange	14.68 ± 2.47	12.36 ± 2.52	< 0.01
Control	14.81 ± 1.93	12.58 ± 2.28	< 0.01
Platelets (cells), mean ± SD			
Plasma exchange	208 ± 95	255 ± 93	0.05
Control	230 ± 84	293 ± 121	< 0.01
pH, mean ± SD			
Plasma exchange	7.42 ± 0.05	7.40 ± 0.05	0.30
Control	7.41 ± 0.07	7.36 ± 0.13	0.02
HCO_3_, mean ± SD			
Plasma exchange	22.03 ± 2.89	25.36 ± 7.34	< 0.01
Control	21.41 ± 2.80	24.00 ± 3.61	< 0.01
pCO2, mean ± SD			
Plasma exchange	34.28 ± 6.37	41.55 ± 13.85	< 0.01
Control	33.95 ± 9.45	46.32 ± 22.27	< 0.01
D dimer (µg/L), median (IQR)			
Plasma exchange	0.95 (0.44–1.87)	1.80 (0.70–4.00)	0.09
Control	1.10 (0.60–2.12)	1.60 (0.80–5.92)	0.10
Fibrinogen (mg/dL), mean ± SD			
Plasma exchange	623 ± 264.30	322.60 ± 128.50	< 0.01
Control	540.20 ± 173.70	396.0 ± 143.90	< 0.01
**Tomographic changes**^b^			
Left lung volume affection (ml), mean ± SD			
Plasma exchange	588 ± 450	505 ± 254	0.47
Control	493.50 ± 314.50	510.20 ± 130	0.30
Right lung volume affection (ml), mean ± SD			
Plasma exchange	568 ± 376	498 ± 302	0.48
Control	542.40 ± 305.70	520 ± 295.40	0.50
Total left lung volume (ml), mean ± SD			
Plasma exchange	1205 ± 524	1111 ± 314	0.30
Control	982 ± 380.10	1100 ± 205.20	0.20
Total right lung volume (ml), mean ± SD			
Plasma exchange	1437 ± 625	1193 ± 361	0.05
Control	1230 ± 364	1350 ± 220	0.50

PLEX, plasma exchange therapy.

^a^5–7 days after treatment.

^b^Evaluated 7–10 days post-PLEX.

For secondary outcomes, PLEX therapy effectively reduced pro-inflammatory mediators and increased lymphocyte count, accompanied with a trend to reduce affected lung volume, without effect on SatO_2_/FiO_2_ indicator or mechanical ventilation requirement (50% vs. 55%, OR0.81, 95%CI 0.279–2.398; p = 0.78) or time to start mechanical ventilation (4.80 ± 3.94 vs 3.23 ± 2.69 days PLEX vs controls (p > 0.05); whereas the control group showed a trend to worsen most of pro-inflammatory and pulmonary indicators (Table 2; Figure 1).

Adverse effects related to PLEX were hypotension episodes in five patients (5/20) that were treated with fluid bolus and/or noradrenaline infusion. Four (16%) patients were diagnosed with secondary bacterial infections after 7 days since the last PLEX: 3 pneumonias, 1 empyema. Gram-negative bacteria were isolated in every case.

## Discussion

In our study, plasmapheresis (PLEX) showed to improve survival from patients with severe COVID-19, as compared with control group. Consistently, several case-series have reported a reduction of 28-days mortality associated with PLEX, which ranges between 10% and 28% in patients with SARS-Cov2, ARDS and cytokine release syndrome^16–18^.

Indeed, one study achieved zero mortality in patients with severe COVID that received 5 sessions of PLEX (0% vs 35%, PLEX vs not PLEX), without adverse events reported^19^. This may be due to the fact that in the group that received PLEX only one patient had severe pneumonia and in the control group 50% of the patients did.

It is possible that the effect of PLEX be mediated by the clearance of pro-inflammatory mediators and toxic biological substances with molecular weights bigger than 15,000 daltons, representing a therapeutic option in patients with hyperinflammatory state secondary to SARS-CoV-2 infection. In our study PLEX was effective to clear pro-inflammatory mediators and to reduce mortality; however, it did not prevent mechanical ventilation, suggesting that additional disorders may underlie pulmonary failure.

A point to highlight is the selectivity of patients with potential benefit from PLEX therapy, which include particular features like severe course of COVID and evidence of cytokine storm syndrome. Similarly, several trials using such indications for PLEX were registered during the present pandemic^16,17,20^.

Interestingly, there is not a current accepted definition for cytokine release syndrome, and whether this syndrome represents an appropriate inflammatory response is still controversial^21^. In one of the first reports on the subject, Mehta et al. suggested the use of the HScore due to the biochemical similarity with the hyper-inflammatory state of secondary hemophagocytic lymphohistiocytosis^22^. Other authors have suggested the cytokine profile with disease severity (IL-6, IL-10, TNF-α, IL-2 and MCP-1)^23^. Nasa et al., proposed an intuitive approach for the diagnosis of cytokine release syndrome, based on clinical data: hypoxemia, organ failure and vasopressor requirement; and biochemical levels: C-reactive protein, ferritin, lactate dehydrogenase, D-dimer and IL-6; and who may benefit from tocilizumab therapy^24^.

In the present study, cytokine release syndrome was considered as IL-6 cutoff value higher than 40 pg/mL, accompanied by elevation of other markers of inflammation and lymphopenia. Previously, Guiaro et al, found that an IL-6 cut-off value higher than 35 pg/mL was associated with increased mortality and higher risk for ICU admission^25^. According to systematic revisions, other biomarkers have been considered to select patients for potential benefits from PLEX therapy; like CRP 132 mg/L (79–168.5), ferritin of 1332 µg/L (1125–1444) and lymphocytes 0.7 × 10^9^/L (0.58–1.0)^26^.

Along clearance of pro-inflammatory mediators, PLEX showed to improve lymphopenia in our study, potentially representing a prognosis modification, since severe lymphopenia (< 500/mm^3^) has been found as independently associated with higher mortality rate in COVID-19 (adjusted OR of 5.63)^27^.

Regarding adverse events, potential mechanisms related to PLEX has been described to clearance of procoagulant factors, immunoglobulins and cytokines, leading to risk of bleeding and/or immunosuppression. In our study only 4 patients developed hospital-acquired infections like pneumonia and one subject developed empyema. Finally, the length of hospital stay was significantly longer in the group with PLEX. Comparatively, PLEX therapy during Faqihi’s trial significantly reduced length of ICU stay. This difference may be explained because institutional CT scan programming was performed at day 10 after the first session of PLEX, independently from severity scores^17^.

Limitations of the present study include a single-center design with a limited sample size that may have caused a statistical overestimation of effect size. In addition, control-case match was uncomplete, which may be explained by the reduced number of candidates for PLEX, who also had incomplete information for adequate analysis. Likewise, the heterogeneity of pharmacologic therapy, which was inherent to the available scientific evidence during the course of pandemia. For example, PLEX and control group received the same non-specific therapy, which included immunoglobulin; but we cannot rule out potential effect of PLEX on immunoglobulin clearance.

## Conclusion

PLEX therapy provided significant benefits of pro-inflammatory clearance and reduction of 60-days mortality in selected patients with COVID-19, without significant adverse events. These results are relevant to better characterize the effect of PLEX in patients with COVID-19; which may contribute to establish more specific therapeutic protocols, based in selection of potential candidates and expected benefits.

## Data Availability

The datasets generated and analyzed during the current study are not publicly available due to privacy policies of the hospital and patients information; but are available from the corresponding author on reasonable request.

## Abbreviations

COVID-19: Coronavirus disease 19
PLEX: Plasma exchange therapy
SARS-CoV-2: Severe Acute Respiratory Syndrome-Coronavirus-2
SOFA: Sequential Organ Failure Assessment
NEWs-2: National Early Warning Score 2
TNFα: Tumor necrosis factor alpha
CRP: C-reactive protein
IL: Interleukin
ARDS: Acute Respiratory Distress Syndrome
PV: Plasma volume
CRRT: Continuous renal replacement therapy
NLV: Non-affected lung parenchyma volume
LOV: Lung opacities volume
OR: Odds ratio
BMI: Body mass index
ESR: Erythro-sedimentation rate
